# Health literacy in female patients affected by anorexia nervosa and bulimia nervosa: a cross-sectional study with pair-matched controls

**DOI:** 10.1007/s40519-023-01588-7

**Published:** 2023-07-13

**Authors:** Chiara Campanino, Andrea Falone, Eleonora Rossi, Lorenzo Lucherini Angeletti, Emanuele Cassioli, Sara Gemignani, Giulietta Brogioni, Giovanni Castellini, Guglielmo Bonaccorsi, Edoardo Mannucci, Valdo Ricca, Francesco Rotella

**Affiliations:** 1 Psychiatric Unit, Department of Health Sciences, University of Florence, Florence, Italy; 2 Department of Health Sciences, University of Florence, Florence, Italy; 3 Diabetes Agency, AOU Careggi Hospital, Florence, Italy; 4 Psychiatric Unit, Careggi University Hospital, Florence, Italy

**Keywords:** Health literacy, Anorexia nervosa, Bulimia nervosa, Eating disorders

## Abstract

**Purpose:**

Health Literacy (HL) consists in all the skills and knowledges used by people to understand and seek health-related information. Inadequate levels of HL substantially affect many different aspects of health. The primary aim of the present study was to assess levels of HL in female patients with anorexia nervosa (AN) and bulimia nervosa (BN), compared with matched control subjects.

**Methods:**

A consecutive series of 64 female patients with AN and BN (mean age 23.1 ± 7.0) was enrolled, matched with 64 female control subjects (mean age 23.7 ± 7.1). Both groups completed the Health Literacy Survey Questionnaire (HLS-EU-Q16) and the Newest Vital Sign (NVS), which evaluate subjective and objective HL level respectively.

**Results:**

Patients with AN and BN showed lower levels of subjective HL (10.0 ± 3.5 vs. 11.3 ± 3.0) and higher levels of objective HL (5.0 ± 1.3 vs. 3.6 ± 1.6) when compared with controls. No difference between AN and BN was found. No correlation between HLS-EU-Q16 Total Score and duration of illness was found. A negative correlation was found between EDE-Q Eating Concerns and subjective HL levels. HLS-EU-Q16 Total Score was predicted by educational level in control subjects only, while NVS Total Score was not predicted by educational level in control subjects nor in patients.

**Conclusion:**

Patients with AN and BN had lower levels of subjective HL. NVS scores could overestimate objective HL in female patients with AN and BN. The promotion of HL in areas differing from those that concern nutritional characteristics of food, could be a therapeutic target for these patients.

**Level of evidence:**

III: Evidence obtained from well-designed cohort or case–control analytic studies.

## Introduction

The term Health Literacy (HL) was first introduced in the 1970s [1] and it can be considered as the development of a specific context of literacy, specifically the one regarding health. Literacy can be defined as “*the ability to identify, understand, interpret, create, communicate and compute, using printed and written materials associated with varying contexts*” [2]. Therefore, for many years HL has been defined as the ability to use words and numbers belonging to the medical world. Recently, the concept has been expanded to a new more complex definition that encompasses different skills and abilities [3]. In 2011, the European Health Literacy Survey was performed to evaluate HL in European citizens. This project led to the development of a further definition of the concept of HL defined as the set of all the skills, knowledges, and competencies that people need to own in order to seek, understand and use health-related information, to express opinions and make decisions about healthcare, disease prevention and health promotion in everyday life and to maintain or improve their quality of life. Individuals with an adequate level of HL should be able to take care of their own health and that of their family and community, but almost a half of the evaluated sample reported insufficient levels of HL [3, 4]. A similar study conducted in Italy in 2016 confirmed these results, as only 40% of the recruited sample showed adequate HL levels [5]. These findings should be considered particularly alarming, since it has been reported that a limited or inadequate HL substantially affects health and it is associated with a lower use of preventive health services [6], greater social inequity [7], riskier habits (such as tobacco consumption) [7, 8], low medication compliance [7], more frequent hospital admissions [7, 9] and premature deaths [7].

To date, no definitive, reliable and comparable HL assessment tool has been proposed for the global population. HL can be assessed by either self-report measures, i.e., those that ask patients a subjective assessment of their skills and knowledges (subjective HL), or functional, performance-based instruments, considered to be objective in their assessment (objective HL) [10]. With respect to objective HL, the Newest Vital Sign (NVS) [11] has been widely used in different populations [12, 13]. It has been reported to be easy and quick to administer, extremely sensible and the most accurate in detecting low levels of HL [14]. Besides, it is easily adaptable for its administration in almost all non-anglophone countries because it is totally retrieved from a nutritional label [15–17].

It is not clear whether self-report and performance-based measures display differences in terms of health outcomes and use of health services, due to the paucity of studies specifically designed to address this topic [10], but it has been hypothesized that the use of both types of questionnaires could improve the accuracy of HL assessment [18].

Eating Disorders (EDs) are complex psychiatric disorders characterized by a common psychopathological core represented by an excessive importance attributed to body shape and weight [19]. Anorexia nervosa (AN) and Bulimia Nervosa (BN) represent a relevant health problem, particularly affecting adolescents and young adults, more frequently females, in industrialized countries [20]. Both AN and BN show drive for thinness and an intense and irrational fear of possible weight gain [21]. AN and BN can often lead to serious physical complications related to malnutrition and improper eating behaviors (including extreme fasting, excessive physical activity, self-induced vomiting, abuse of diuretics and/or laxatives). For this reason, they are burdened by a significant mortality risk [21–23].

Since AN and BN are characterized by the presence of excessive concerns about weight and body shape that lead to a strict control over nutrition through cognitive and/or caloric restriction, patients usually acquire a considerable knowledge of health and healthy behaviors, as well as nutrients’ composition of food and of its caloric content [19, 24, 39]. At the same time, eating psychopathology may affect the ability to understand and use health information effectively. It has been reported that patients with EDs may have difficulties in understanding and following instructions for a healthy diet, or in recognizing body signals indicating hunger or satiety [25, 26]. It could be therefore hypothesized that low levels of HL may be linked to ED psychopathology, affecting the way individuals understand and integrate the effects of their eating behaviors on their health. More in detail, lower levels of HL could be associated to more severe eating disordered behaviors, such as counting consumed and burned calories and estimating food weight and composition. On the other hand, the NVS, which is an instrument entirely based on a nutritional label, may overestimate HL levels in subjects with EDs.

To the best of our knowledge, HL has been never evaluated in patients with EDs. Our hypothesis was that EDs subjects display lower levels of HL. However, the administration of NVS as assessment tool for objective HL, could produce a distortion of the results, showing similar, or even higher, levels of objective HL in female patients with AN and BN, compared to matched control subjects.

Therefore, the primary aim of the present study was to evaluate subjective and objective HL in patients with AN and BN, compared to a group of control subjects matched for sex, age and educational level.

Secondary aims were to (1) compare the levels of objective and subjective HL between patients with AN and BN; (2) evaluate the possible effect of duration of illness on HL among patients; (3) evaluate the possible correlation between psychopathology core of EDs and HL levels; (4) evaluate the relationship between educational levels and HL both in patients and control subjects.

## Methods

A cross-sectional observational study with pair-matched controls was conducted.

### Ethical statement

Patients and controls were informed about the study procedures and signed informed consent to participate. In case of people aged under 18 years old, the informed consent was signed by legal representative. The study protocol was accepted and authorized by the local ethics committee (Ethical Committee Area Vasta Centro, approval number of the protocol: 16069).

### Participants

A power analysis was performed using G*Power v3.1.9.2 [27], and a total sample size of 128 (64 participants for each group) was determined to be adequate to detect a medium effect size (*d* = 0.5) [28] using independent samples t-test (power = 0.80, *α* = 0.05).

A consecutive series of patients referring to the outpatient clinic of the Psychiatry Unit of Careggi Teaching Hospital was included, provided that they fulfilled the following inclusion criteria: female sex, age over 16 years old, current diagnosis of AN or BN according to DSM 5 criteria accomplished by an expert psychiatrist [19]. Exclusion criteria were: comorbid schizophrenia or bipolar disorder, illiteracy and intellectual disability.

Control subjects (1:1) were recruited by spreading the study protocol in the university premises and by social media advertisements among students of the University of Florence, extending the invitation to siblings, friends and other acquaintances of students fulfilling the inclusion criteria. Inclusion criteria were as follows: female sex, age over 16 years old. Exclusion criteria were as follows: illiteracy, intellectual disability, a self-reported previous diagnosis of schizophrenia, bipolar disorder or EDs.

### Assessment and measures

Sociodemographic and clinical data of all the study participants were collected, including gender, age, educational level, weight, height (using calibrated instruments for patients and self-reported measures for controls) and computed body mass index (BMI). For patients, data on duration of illness were also collected. Moreover, the following questionnaires were administered:

- Health Literacy Survey Questionnaire (HLS-EU-Q16) [29], to evaluate subjective HL levels. This is a self-administered questionnaire composed of 16 questions investigating skills and abilities in order to assess HL in three different domains (health care, disease prevention, health promotion). The questionnaire requires assessing the level of difficulty in accessing, understanding, evaluating and applying health-related information using a scale from extremely difficult to extremely easy. The final score is calculated by dividing the answers into two categories, “easy” (“easy” or “very easy” = 1) and “difficult” (“difficult” or “very difficult” = 0). The answer "I don't know" is calculated as missing. The final score results from the sum of each item answer and ranges from 0 to 16, so identifying three levels of HL: inadequate (0–8), problematic (9–12) and adequate (13–16).
- Newest Vital Sign (NVS) [11]: hetero-administered questionnaire that evaluates objective HL levels. It consists in submitting to the person's attention an ice-cream nutritional label (e.g. the one that could be bought at the supermarket), asking him seven questions about it. This questionnaire measures general literacy and the ability of reading, reasoning and using numbers (numeracy) rather than considering all the aspects included in the definition of HL. The final score ranges from 0 (minimum) to 6 (maximum): people who answer correctly 0–1 questions are more likely (50% or more) to have a limited HL; people who answer correctly 2–3 questions have the possibility of limited HL; while, a score of 4 to 6 shows adequate HL.
- Eating Disorder Examination Questionnaire (EDE-Q) [30]: self-reported questionnaire which evaluates eating psychopathology and its related pathological behaviors. The questionnaire is composed by 22 items, where higher scores indicate a greater severity of eating psychopathology. Each item scores from 0 to 6. Other 6 items investigate the number of pathological eating behaviors, such as binge eating, use of laxatives, self-induced vomiting and excessive exercise, in the last 28 days. The scale provides a global score based on four subscales (restraint, food concern, body shape concern and weight concern) which reflect the severity of the psychopathological traits of EDs. The global score is obtained by the mean score of the four subscales. Only the group of patients completed this questionnaire.

A questionnaire was considered valid when more than 85% of the items were adequately completed.

### Statistical analysis

Continuous variables were reported as mean and standard deviation. Comparisons of levels of subjective and objective HL between the two matched groups were performed by means of independent samples *t-tests*. Furthermore, subjective and objective HL levels were compared between diagnostic groups (AN and BN) using *t-tests*. Pearson’s correlation coefficients were computed to assess the correlations of HLS-EU-Q16 and NVS scores with age and education in both groups, and with duration of illness and ED psychopathology in the group of patients only. In order to investigate whether the relationship between NVS and HLS-EU-Q16 with age and education was different in patients compared to the control group, moderation analysis was performed using linear regression models in which the presence or absence of an ED and its interaction with the independent variable were entered as predictors. Significant interaction effects were probed using simple slope analysis. All statistical analyses were performed using R [31].

## Results

A consecutive series of 69 patients was invited to participate between November 2019 and May 2021. Of the 69 patients invited, 5 denied participation or failed to complete the questionnaires, therefore the final sample consisted of 64 patients (43 patients with AN and 21 with BN). Sixty-four control subjects, matched for sex, age and educational level with patients at a ratio of 1:1, were selected from a total of 205 subjects contacted.

All patients enrolled were females. Table 1 shows the main demographic and clinical characteristics of the two samples. Patients with EDs reported lower levels of subjective HL and higher levels of objective HL compared to control group (Table 1). No statistically significant differences emerged between diagnostic groups comparing levels of HLS-EU-Q16 Total Score (AN group: *mean* = 9.86, *SD* = 3.49; BN group: *mean* = 10.38, *SD* = 3.56; *t* = − 0.56, *p* = 0.58) and NVS Total Score (AN group: *mean* = 5.09, *SD* = 1.17; BN group: *mean* = 4.86, *SD* = 1.49; *t* = 0.69, *p* = 0.49).

**Table 1:** Main characteristics of the sample

	Patients (n = 64)	Control subjects (n = 64)	t
Age	23.14 ± 7.04	23.69 ± 7.13	− 0.44
BMI	18.40 ± 4.39	21.61 ± 2.82	− 4.82***
Education	13.50 ± 2.47	13.48 ± 2.44	0.036
Illness duration	6.47 ± 7.26		
HLS-EU-Q16 total score	10.03 ± 3.49	11.30 ± 2.99	− 2.20*
NVS total score	5.02 ± 1.28	3.61 ± 1.61	5.47***

Data reported as mean and standard deviation, with the comparison between patients and controls performed with Student’s t-test

*BMI* Body Mass Index, *HLS-EU-Q16* Health Literacy Survey Questionnaire, *NVS* Newest Vital Sign

*p < 0.05; ***p < 0.001

Correlations of HLS-EU-Q16 and NVS scores with age, education, duration of illness and psychopathology in patients are reported in Table 2. No significant correlation between levels of HL and duration of illness was found (Table 2), whereas subjective HL showed a significant negative correlation with EDE-Q Eating Concerns scores (Table 2). No other statistically significant correlation was found between HLS-EU-Q16 or NVS scores and ED psychopathology (Table 2). Considering control subjects, a significant positive correlation was found between educational level and HLS-EU-Q16 (r = 0.35, p = 0.005), whereas no significant correlation was found between levels of subjective and objective HL and age.

**Table 2:** Correlations of HLS-EU-Q16 and NVS scores with age, education, duration of illness and psychopathology in the group of patients

	NVS	HLS-EU-Q16
Age	− 0.08	0.18
Educational level	0.18	− 0.15
Duration of illness	− 0.08	0.20
EDE restraint	0.19	− 0.17
EDE_Eating concern	0.17	− 0.28*
EDE_Weight Concern	− 0.03	− 0.2
EDE_Shape concern	0.06	− 0.22
EDE total score	0.1	− 0.23

*p < 0.050

The association between subjective HL and educational level was moderated by the presence of an ED, as evidenced by a significant Education*Group interaction effect (*b* = − 0.64, *p* = 0.01). Simple slope analysis revealed that HLS-EU-Q16 Total Score was significantly predicted by educational level in control subjects only (*b* = 0.42, *p* = 0.01), but not in patients with EDs (*b* = − 0.21, *p* = 0.19). Conversely, the relationship between the level of objective HL and educational level was similar in control subjects and patients (*b*_*Education*_ = 0.14, p = 0.05) with a non-significant Education*Group interaction effect (b_Education*Group_ = − 0.05, *p* = 0.617).

Moderation analysis confirmed the absence of a correlation between age and HLS-EU-Q16 Total Score (*b*_*Age*_ = 0.05, *p* = 0.384; *b*_Age*Group_ = 0.04, *p* = 0.613) and between age and NVS Total Score (*b*_*Age*_ = 0.02, *p* = 0.439; *b*_Age*Group_ = − 0.04, *p* = 0.327).

Lastly, HLS-EU-Q16 and NVS did not show any statistically significant correlation with each other in the whole sample (r = 0.07, p = 0.449), nor in the control group (r = 0.15, p = 0.238) or the group of patients (r = − 0.12, p = 0.341).

## Discussion

Patients with AN and BN showed higher levels of objective HL and lower levels of subjective HL than matched controls.

NVS represents a widespread tool used to assess levels of objective HL [13], but it seems to be not adequate for all populations [32–34]. The discrepancy between levels of subjective and objective HL found in the present study allows to speculate that this tool overestimates objective HL levels in female patients with AN and BN. Further studies are needed in order to evaluate levels of objective HL in subjects with EDs, using different tools.

Patients with AN and BN usually display more nutritional knowledges than healthy people as they are familiar with calories, macronutrients and energy expenditure [35]. Higher levels of objective HL are therefore consistent with the fact that patients with AN and BN try to exercise an extreme control over the caloric and nutritional content of foods through cognitive and/or caloric restriction in response to an intense fear of gaining weight and the consequent change in body shapes [19, 39].

For this reason, an accurate assessment of objective HL appears to be extremely relevant in this population. Despite being classified as rare disorders, the prevalence of EDs is increasing, particularly among young women [36, 37]. A recent study estimated that point ED prevalence increased from 3.5% for the 2000–2006 period to 7.8% for the 2013–2018 period [37]. Furthermore, it is believed that the prevalence of EDs may be underestimated as not all types of sub-threshold EDs are included in most epidemiological studies [38]. Additionally, many prevalence studies use self-report tools to assess and diagnose EDs and are retrospective, leading to memorization biases and to an incomplete representation of the true current prevalence estimates [37]. Consequently, our data could suggest that a significant portion of general population (e.g., young females) may not be adequately evaluated for objective HL levels, using NVS.

The low subjective HL shown by patients suffering from AN and BN, might represent the evidence that these subjects do not have adequate levels of HL regarding broader aspects of health (e.g., understand and use health-related information, or health promotion in everyday life). Indeed, the psychopathological core of these disorders make body shape and weight the main focus of daily living with a consequent marginalization of all other aspects of life, including social and intimate relationships, school, and work [39]. Therefore, it is not surprising that patients tend to put aside everything that does is not strictly related to this context. In particular, patients with AN usually suffer from social withdrawal, having little social interactions and few interests in their lives [40]. These features, associated to low levels of self-efficacy and self-esteem often reported in AN and BN [41, 42], could represent the reasons why these patients consider their ability to deal with the health system as insufficient. Furthermore, patients with AN suffer from a low insight of illness and they often tend to deny their disease as much as possible [43] and avoid interactions with health system.

As an overall, HL could represent an important target for both prevention and therapeutic programs in female patients with AN and BN, as higher HL levels could be associated to lower incidence and progression of an eating disorder, or to a better outcome. Longitudinal studies and randomized-controlled trails are needed to better investigate this issue.

In the present study, HL levels were similar in the groups of patients with AN and BN, in line with the existence of common psychopathological features between these two disorders [39]. However, the study was not specifically designed to assess differences between patients with AN and BN in terms of HL levels and the two cohorts (AN and BN) were not matched. Therefore, results of comparisons between patients with AN and BN should be considered merely exploratory.

This study also suggests that HL is not affected by the duration of illness in female patients with AN and BN. It can be hypothesized that an increase in objective HL is acquired early in the natural history of EDs and maintained afterwards regardless of the clinical evolution of the disorder. It is possible that such trait is developed in pre-clinical phase, when a formal diagnosis of AN or BN cannot be formulated yet. This may explain also the absence of correlation between eating psychopathology and objective HL levels. The computational skills displayed by patients with AN and BN, assumed to be associated to higher objective HL levels, could be considered as a trait condition, not affected by the severity of symptoms. On the other hand, the negative correlation found between eating concerns and subjective HL levels suggests that the more the eating concerns are high, the more the capacity of these patients to interact with health system decreases.

Educational level affected objective HL levels both in patients and controls, in line with previous studies [5, 29]. Conversely, subjective HL levels were associated with educational level in control group only, but not in patients. The intrusive psychopathological core that characterizes EDs, which promotes their isolation from everything that does not strictly concern nutrition, weight and body shapes, can affect their capacity to access to the health system, independently from their education.

Lastly, no correlation was found between NVS and HLS-EU-Q16 questionnaires, as already shown in literature [29]. This result could derive from the fact that these questionnaires evaluate different aspects of the construct of HL (objective vs. subjective evaluation). Therefore, the use of both of these questionnaires seems to help better investigating the concept of HL in individuals [18]. In light of this, as already stated, it could be interesting to evaluate objective HL levels in patients with AN and BN with a further questionnaire which do not refer to nutrition and food only, possibly overestimating objective HL levels in this way.

## Strength and limits

Some limitations should be considered when interpreting results of the present study. All patients have been enrolled in a single specialist Outpatient Clinic, therefore they cannot be considered representative of the whole population of patients with EDs. Furthermore, control subjects were enrolled on the basis of a voluntary response to a general invitation to participate in a study about EDs, possibly selecting subjects with higher HL levels, specifically in the area of nutrition. In addition, the sample was relatively homogeneous for age, possibly preventing the observation of differences with respect to this parameter. It should also be considered that control subjects self-reported weight and height, so that the mean BMI obtained in the control group may be under-estimated, as already stated in literature [44]. Lastly, the assessment of ED psychopathology in control group was evaluated through self-report of past ED diagnoses and BMI.

To our knowledge, this is the first study evaluating HL in patients with EDs. Another important strength is represented by the simultaneous assessment of subjective and objective HL, that allowed a comparison between the results obtained from the assessment of the two different dimensions.

## What is already know on this subject?

HL is a complex and multidimensional concept that has been widely explored over the last years. Individuals with an adequate level of HL should be able to take care of their own health and that of their family and community. Epidemiologic surveys conducted on this topic showed that almost a half of the evaluated samples reported insufficient levels of HL.

## What this study adds?

Patients with EDs show lower subjective and higher objective HL levels than matched controls. NVS seems to overestimate levels of HL in EDs population. The promotion of HL in specific areas, excluding those that concern nutritional characteristics of food, could be a prevention and/or therapeutic target for EDs.

## Data Availability

Research data are not shared.
